# Women's empowerment, extended families and male migration in Nepal: Insights from mixed methods analysis

**DOI:** 10.1016/j.jrurstud.2022.01.003

**Published:** 2022-02

**Authors:** Cheryl R. Doss, Ruth Meinzen-Dick, Audrey Pereira, Rajendra Pradhan

**Affiliations:** a Professor of International Development in the Department of International Development, University of Oxford, Oxford, UK; b Senior Research Fellow in the Environment and Production Technology Division of International Food Policy Research Institute, USA; c PhD Student in the Department of Public Policy at the University of North Carolina at Chapel Hill, USA; d Managing Director of the Nepā School of Social Sciences and Humanities, Kathmandu, Nepal

**Keywords:** Gender, Caste/Ethnicity, Joint families, Migration, Time use, Women’s Empowerment in Agriculture Index

## Abstract

• Combining qualitative and quantitative methods sheds new light on women's empowerment processes.
• Upper caste Nepali women are disempowered by patriarchy; lower caste women by poverty and patriarchy.
• Non-migrant husbands mediate the disempowering effects of living with in-laws.
• Control over time, not just hours worked, is an important component of empowerment.

## 1 Introduction

Recognition of the importance of women's empowerment, for both intrinsic and instrumental reasons, prompts researchers and development practitioners to seek understanding of the factors that affect empowerment. Although much of the literature on empowerment has been qualitative, increasingly quantitative indicators are being used to measure empowerment, often to assess the impact of development interventions (see Alkire et al., 2013; Malapit et al., 2019; Yount et al., 2016). Bringing both qualitative and quantitative methods together is an important means of validating empowerment measures (Jayachandran et al., 2021). But tensions and even contradictions between emic and etic perspectives that emerge from qualitative and quantitative findings can help us to deepen our understanding of women's empowerment.

Many efforts to measure empowerment have focused on the spousal couple, ignoring the effect of extended families and the dynamics of changes over the life cycle. In the South Asian context, and Nepal in particular, extended family structures, or joint households, have long been associated with disempowerment of daughters-in-law in the ethnographic literature (Desai and Banerji 2008; Rashid 2013; Kaspar 2005; Adhikari and Hobley 2015; Singh 2016; Sijapati et al., 2017; see also Allendorf 2007 and Rajkarnikar 2017, 2020 for quantitative analysis of this issue). As we shall show, social position in the household, as a mother-in-law or daughter-in-law or wife of a male head of household, is an important factor influencing empowerment.

Migration patterns further complicate household dynamics and life cycles, and the effect on women's status and ability to make strategic life choices is not clear. Pervasive male emigration from rural Nepal can increase the sphere of women's decision-making and control over resources, but it may also place additional burden on their time, thereby restricting their agency (Kaspar 2005; Maharjan et al., 2012; Sijapati et al., 2017). Thus, the impacts of male migration on women's empowerment are ambiguous.

Another important factor which influences empowerment is caste and ethnic identity because caste/ethnicity often influences norms pertaining, for example, to physical mobility, paid labour, and marriage, which in turn affect empowerment.

To examine how social position in the household interacts with male migration as well as caste and ethnicity to affect both emic and etic perceptions of empowerment, we use both qualitative and quantitative data from an impact assessment of a livestock transfer project in Nepal (Janzen, Magnan and Thompson 2018). In an earlier analysis of the qualitative data, Pradhan et al. (2019) identify factors affecting women's property rights through in-depth life histories and focus groups. They find that social location within the household plays a significant role, with women's empowerment changing across the life cycle. Daughters-in-law report having few property rights, particularly compared to their mothers-in-law; women's property rights significantly increase when the household splits and she becomes the co-head of household. Although the study identified differences in norms and practices regarding property rights for different castes and ethnic groups, it was not possible to identify the relative effects of caste, ethnicity, and household structure on this aspect of women's empowerment. However, the project also has quantitative data on other domains of empowerment that are included in the Abbreviated Women's Empowerment in Agriculture Index (A-WEAI), as well as qualitative data on these and other aspects of empowerment. We use this data to examine the relationships of caste or ethnicity, women's social location in the household, and husband's migration status with women's empowerment.

We first discuss concepts of empowerment and the factors that are likely to affect women's empowerment, based on a review of the conceptual and empirical literature. We then present the methods and data. In presenting the results, we begin with an overview of the factors affecting the overall empowerment score, composed of six indicators, from the A-WEAI. Subsequent sections provide a detailed qualitative and quantitative analysis of how each factor affects individual indicators: input in productive decisions, asset ownership, access to and decisions on credit, control over use of income, group membership, and workload.

By bringing together qualitative and quantitative data from the same sites, our study makes three key contributions to the understanding of women's empowerment. First, we find that women's empowerment is strongly associated with caste/ethnic identity, but not necessarily in the expected ways. Whereas much of the ethnographic literature, including our qualitative findings, emphasizes stronger patriarchal norms placing restrictions on high-caste women, our quantitative results show greater disempowerement among low-caste (Dalit) and other ethnic groups (Janajati), and Terai middle castes, indicating that disempowerment may come from poverty, as well as patriarchy.

Second, our quantitative results also provide insights regarding the relationship of position in the household and empowerment. While qualitative analyses often stress the disempowerment of daughters-in-law, our quantitative findings suggest that having a resident husband, as opposed to a migrant husband, can mediate this disempowerment. In contrast, wives in nuclear households are more likely to be empowered when their husbands are migrants. Thus, husbands play a mediating role, particularly when the couple lives with his parents—a role that is under-explored in the literature.

Finally, the contrast between qualitative findings that daughters-in-law feel overworked and quantitative findings that show no statistically significant difference in hours worked based on social location within the household, suggests that we need to rethink how time use affects empowerment, focusing on agency over time and intensity of work rather than simply on hours worked, to more accurately reflect emic, as well as etic, understandings of empowerment.

### 1.1 Conceptual foundations

While there are numerous definitions of empowerment, many follow Kabeer’s (1999) focus on processes of acquiring the ability to make strategic life choices. O'Hara and Clement (2018) point out the limitations of applying standardized definitions of empowerment that focus on visible forms of agency, and make the case for attention to critical consciousness (Freire 1970), or “power within” (Rowlands 1995). It is also valuable to go beyond etic definitions and analyses, to understand emic definitions and localized understandings of empowerment processes (Meinzen-Dick et al., 2019). Nor is empowerment static: it changes over the life cycle and is affected by social location within the household and whether a woman's husband has migrated or is resident in the household. Finally, wealth and other socioeconomic characteristics also play an important role. It is therefore essential to consider various dimensions of intersectionality to understand empowerment processes (Carr and Thompson, 2014; Colfer et al., 2018).

Nepal provides a good case study for exploring intersectionality and what we can learn about women's empowerment from using mixed methods approaches. In Nepal, caste and ethnicity are particularly relevant to understanding empowerment. As elsewhere in South Asia, Nepal's caste system is very hierarchical. Brahmins occupy the highest status, closely followed by Chhetris (equivalent to Kshatriya in the varna system). Dalits are the lowest castes in Nepal and have faced pervasive discrimination and exclusion. There are also numerous ethnic groups, especially in the hill areas, who fall outside the strict limits of the Hindu caste hierarchy, and who also have faced discrimination from the upper caste Hindus.

In mainstream Nepali culture, adherence to patriarchal norms such as restrictions on women's public voice, marriage choice, divorce, freedom of movement, and employment is associated with prestige. Gender norms are considered more egalitarian among most ethnic groups (Janajati) and Dalit castes than among higher Hindu castes (Acharya and Bennett 1983; Kaspar 2005; Adhikari and Hobley 2015; Rajkarnikar 2017; Sijapati et al., 2017), particularly in the lack of prohibitions against divorce and remarriage. There is, however, very little difference from high castes in gender relations in other important areas such as inheritance, political and ritual authority (Pradhan 2014; Shah 2018).

These findings do not mean that Dalit or Janajati women are necessarily empowered. While they are less restricted by gender norms on marriage or mobility compared to high-caste Hindu women, the intersectionality of caste and class means that Dalit or Janajati women are often disempowered by poverty, exclusion and social stigma affecting their whole household or social group, limiting their access to credit and participation in local groups. Most ethnic groups and Dalits are poorer and have lower Human Development Index than the upper castes (Government of Nepal and UNDP United National Development Program, 2014; see also World Bank/DFID 2006; Bennett 2008; Bennett et al., 2013). Because of their poverty, women from these groups are forced to work for others to earn money for the family. While this may give them greater decision-making power within their household and ability to travel within communities, the work is often low-status and increases their overall workloads (Sugden et al., 2014). While higher caste women are often disempowered by gender norms relative to the men in their households, Tamang (2011) notes that they have benefitted from caste-and family-specific privileges and access to education, while lower class Dalit and Janajati women and men are disempowered in other key areas by poverty and social exclusion that limit their access to education, resources, and opportunities (Clement et al., 2019; Satyal Pravat 2013).

Social location within the household provides another critical factor in determining the status of women in any society, but particularly in South Asia, where joint families are common. Pradhan, Meinzen-Dick and Theis (2019) describe a broad process in which Nepali women (daughters-in-law) living with their husband's parents and siblings are the most disempowered; women become more empowered when their household splits off and they are identified as a “wife”-- the co-head of a nuclear household. Mothers-in-law are perceived as the most empowered, because they have “power over” daughters-in-law, but women again become vulnerable as they age, especially when they become widows and worry about who will care for them.

Widespread migration in Nepal, especially of men, further affects women's empowerment. Migration within rural areas of Nepal, to urban centers, and across the border to India may be short-term, with migrants returning for key events such as harvest. Long-distance international migration to the Middle East and Malaysia is usually for longer periods. A number of studies have noted increases in women's mobility and decision-making roles after their husbands migrate, especially among Dalit and Janajati households (Kaspar 2005; Sijapati et al., 2017). However, wives with migrant husbands have stress from added responsibilities and workload, both domestic/care and especially productive non-domestic work, which can lead to disempowerment (Kaspar 2005; Adhikari and Hobley 2015; Sijapati et al., 2017; Rajkarnikar 2017; Clement et al., 2019; Clement and Sugden 2021).

While each of these factors—gender, caste or ethnicity, household structure, and migration—affects women's empowerment, they are not experienced individually, but interact in many different ways. Because of this complexity, there are relatively few studies that deal with the intersection of these aspects. In this paper, we allow quantitative and qualitative data to interrogate each other, to gain a better understanding of the relationships among these four factors affecting empowerment.

## 2 Methods

### 2.1 Data sources

Qualitative and quantitative data come from an impact assessment of Heifer International's Smallholders in Livestock Value Chain Program in Nepal (Janzen et al., 2016, 2018).^1^ Quantitative analyses use the 2016 midline survey of Heifer International's impact evaluation, with a total sample of 1803 women from 50 Village Development Committees. They are from five districts: Tanahu, Palpa, Rautahat, Sarlahi, and Mahottari.

The qualitative research was conducted in four villages that were part of Heifer International's livestock transfer programs. Villages were purposively selected to include two in the hills and two in the Terai. To examine the effect of caste and ethnicity, sites were selected to include four ethnic groups: Tamang (in Kafaltar, Dhading), Magar (in Arbasing, Palpa), Tharu (in Patwari, Nawalparasi) and Madhesi (in Kisannagar, Mahottari).

Two-person field research teams fluent in the local languages and trained in ethnographic methods by Nepā School of Social Sciences and Humanities were resident in the villages for 60 days conducting direct observation, semi-structured interviews, life-histories and focus group discussions on topics of empowerment, social capital, property rights, and migration. Attempts were made to select respondents from different ethnic groups, castes (especially Dalits), members of nuclear and of joint households, women from migrant and non-migrant households, and young as well as old women. A total of 188 respondents (148 women) were interviewed. Meetings among the research teams identified the importance of social location and household structure as important factors affecting women's empowerment,illustrated by cases observed of recent household splits. In our presentation of results, we refer to the qualitative findings along with the quantitative results, noting areas of agreement, where the qualitative data provides insights on the underlying reasons for the quantitative findings, as well as divergence where the two methods seem to be telling different stories.

### 2.2 Quantitatively measuring empowerment

This study uses components of the A-WEAI to measure empowerment. The tool, which employs the Alkire-Foster methodology, consists of six indicators: input in productive decisions, asset ownership, access to and decisions on credit, control over use of income, group membership, and workload (Alkire et al., 2012; Appendix A; Malapit et al., 2017). Adequacy in each indicator is defined as meeting or exceeding a pre-determined threshold for that indicator. A woman is considered empowered if she is adequate in at least 80 percent of the weighted indicators.^2^

### 2.3 Indicators of caste/ethnicity, social location, and migration status

For our quantitative analysis, we have grouped the 37 individual castes/ethnic groups/religious groups reported among the survey population, into six broad categories: Brahmin/Chhetri (also referred to as upper castes), Dalit, Janajati, Muslim, Tarai middle castes (Madhesi), and other.^3^

In terms of social location in the household, wives were defined as the co-head of a nuclear household, without resident in-laws. Mothers-in-law lived with at least one daughter-in-law, and daughters-in-law lived with at least one parent-in-law (i.e. mother-in-law or father-in-law). Unmarried daughters living with their parents were not included in our analysis; they were less than two percent of the entire sample and could not be included in the analysis of husband's residence status.

Husbands are defined as migrants if they are away from the household on domestic or international, short- or long-term migration. Although we would expect men on long-term, international migration to be less involved in household management than domestic, short-term migrants, the categories are not easily distinguishable in practice. Crossing the border to India from the Terai would qualify as international migration, although it may be closer than migration to Kathmandu. Short-term migration, defined in the survey as those who have been away for less than six months, includes those who plan to be away for several years. Thus, our analysis does not distinguish between types of migrants.

### 2.4 Empirical strategy

We are interested in the relationship of caste/ethnicity, women's social location in the household, and whether her husband is a migrant, with her level of empowerment. The basic model that we estimate is:

$$Y_{i}=\alpha +\mu X_{i}+\beta Social\;location_{i}+\gamma Migrant\;husband_{i}+\delta Social\;location_{i}\cdot Migrant\;husband_{i}+\;\varepsilon_{i}$$

where Yi are the empowerment outcomes: (i) empowerment score, calculated as the weighted sum of the six binary indicators, and (ii) adequacy in each of the six indicators.^4^ X_i_ is a set of individual- and household-level characteristics, including caste/ethnicity (reference: Brahmin/Chhetri), age, years of schooling, number of children under five years old in the household, number of children age 5–18 in the household, number of adult women and men in the household, and an asset index.^5^ Social location is defined as being either a wife (reference), mother-in-law, or daughter-in-law. Migrant husband is a dummy variable indicating whether the husband is in residence (reference) or currently a migrant. The effect of empowerment on varying levels of social location and husband's migration status is captured by δ. We initially estimate the empowerment score regressions using Ordinary Least Squares (OLS), then unpack the various domains of empowerment using logistic regressions for the six binary A-WEAI indicators. All analyses account for complex survey design.^6^

## 3 Results

### 3.1 Descriptive characteristics of the sample

Descriptive characteristics and t-tests (reference: Wife; Husband in household) are presented in Table 1. The majority of the sample consisted of Dalit (36%), followed by Brahmin/Chhetri (25%), Terai Middle (20%) and Janajati women (11%). Muslim women and women of other castes/ethnicity each comprised four percent of the sample.^7^

**Table 1:** Descriptive statistics of women in the sample (means and standard errors).

	Wife; Husband in HH	Wife; Migrant husband	Mother-in-law; Husband in HH	Mother-in-law; Migrant husband	Daughter-in-law; Husband in HH	Daughter-in-law; Migrant husband	All women
*Caste (%)*							
Brahmin or Chhetri	0.26 (0.04)	0.22 (0.05)	0.25 (0.04)	0.16 (0.06)	0.27 (0.04)	0.27 (0.05)	0.25 (0.04)
Dalit	0.33 (0.04)	0.40 (0.06)	0.37 (0.05)	0.42 (0.11)	0.33 (0.05)	0.39* (0.06)	0.36 (0.04)
Janajati	0.13 (0.02)	0.10 (0.03)	0.12 (0.02)	0.19 (0.07)	0.09 (0.03)	0.10 (0.03)	0.11 (0.02)
Muslim	0.04 (0.01)	0.03 (0.01)	0.03 (0.01)	0.00***	0.04 (0.02)	0.03 (0.01)	0.04 (0.01)
Tarai Middle	0.21 (0.03)	0.21 (0.04)	0.20 (0.04)	0.23 (0.07)	0.21 (0.04)	0.17 (0.04)	0.20 (0.03)
Other castes	0.04 (0.01)	0.04 (0.02)	0.03 (0.01)	0.00***	0.06 (0.02)	0.03 (0.01)	0.04 (0.01)
*Other factors*							
Age (years)	42.19 (0.75)	34.27*** (0.53)	52.71*** (0.53)	43.58 (1.09)	30.21*** (0.58)	28.60*** (0.47)	39.45 (0.53)
Years of schooling	2.04 (0.20)	3.35*** (0.30)	0.47*** (0.10)	1.10** (0.39)	5.08*** (0.41)	5.77*** (0.34)	2.89 (0.18)
Number of adult women in HH	1.26 (0.03)	1.14*** (0.02)	2.67*** (0.05)	2.65*** (0.17)	2.36*** (0.05)	2.33*** (0.05)	1.88 (0.03)
Number of adult men in HH	1.50 (0.03)	1.30*** (0.04)	2.92*** (0.05)	2.77*** (0.15)	2.33*** (0.06)	2.29*** (0.07)	2.03 (0.03)
Child under 5 years lives in HH	0.18 (0.02)	0.25** (0.03)	0.48*** (0.03)	0.65*** (0.08)	0.47*** (0.03)	0.51*** (0.03)	0.35 (0.02)
Child aged 5–18 years lives in HH	0.73 (0.02)	0.91*** (0.02)	0.75 (0.03)	0.87** (0.06)	0.83*** (0.02)	0.80** (0.03)	0.79 (0.01)
Asset index	−0.29 (0.07)	−0.34 (0.06)	0.27*** (0.06)	0.06*** (0.13)	0.19*** (0.09)	0.23*** (0.07)	−0.03 (0.05)
*Empowerment outcomes*							
Empowerment score (0–1)	0.79 (0.01)	0.82*** (0.01)	0.78 (0.01)	0.85* (0.03)	0.81** (0.01)	0.79 (0.01)	0.80 (0.01)
*A-WEAI indicators (binary)*							
Input in productive decisions	0.92 (0.01)	0.95** (0.01)	0.96*** (0.01)	1.00***	0.93 (0.02)	0.96** (0.02)	0.94 (0.01)
Access to and decisions on credit	0.38 (0.02)	0.44 (0.04)	0.28*** (0.03)	0.52 (0.10)	0.36 (0.03)	0.34 (0.04)	0.36 (0.02)
Control over use of income	0.93 (0.02)	0.98*** (0.01)	0.94 (0.02)	1.00***	0.94 (0.02)	0.96* (0.01)	0.95 (0.01)
Asset ownership	0.95 (0.01)	0.99*** (0.01)	0.96 (0.01)	1.00***	0.93 (0.01)	0.95 (0.02)	0.96 (0.01)
Group membership	0.67 (0.03)	0.76** (0.04)	0.65 (0.03)	0.71 (0.07)	0.78*** (0.03)	0.71 (0.03)	0.71 (0.02)
Workload	0.65 (0.03)	0.59* (0.04)	0.61 (0.04)	0.68 (0.11)	0.67 (0.04)	0.60 (0.05)	0.63 (0.03)
*Time use*							
Time spent on work (total)	8.88 (0.26)	9.24 (0.37)	8.96 (0.28)	8.34 (0.72)	9.01 (0.26)	9.09 (0.26)	8.99 (0.22)
Productive non-domestic work (hours/day)	4.25 (0.26)	4.60 (0.38)	4.94*** (0.28)	4.69 (0.85)	4.13 (0.30)	4.54 (0.34)	4.47 (0.26)
Domestic/care work (hours/day)	4.66 (0.16)	4.69 (0.22)	4.02*** (0.18)	3.83 (0.51)	4.90 (0.21)	4.64 (0.25)	4.56 (0.13)
*N*	605	254	371	31	307	235	1803

Note: Estimates are means with corresponding standard errors clustered at the VDC level in parentheses. Social locations are defined: Wife: no in-laws in the household; Mother-in-law: at least one daughter-in-law in the household; Daughter-in-law: at least one parent-in-law in household. The empowerment score is the weighted average of the six binary A-WEAI indicators provided in Appendix A. Reference category for t-tests is ‘Wife with husband in HH’; t-tests not conducted for the ‘All women’ category. * = p < 0.10; ** = p < 0.05; *** = p < 0.01. Domestic/care work includes time spent on shopping/getting services, weaving/sewing/textiles, cooking, domestic work, caring for children, and caring for adults.

Of the married women in our sample, 48% were wives, 30% were daughters-in-law, while only 22% were mothers-in law. For 29% of the women, their husbands were migrants and not living in the household at the time of the survey. Many more of the daughters-in-law (43%) had migrant husbands, while only 7% of the mothers-in-law did so. As expected, daughters-in-law are both the youngest group and the best educated, although their average education was only 5 years for those with resident husbands and 5.8 years for those with migrant husbands. Compared to wives (who live in nuclear households), mothers-in-law and daughters-in-law lived with more adults in their (extended) households.

Overall mean empowerment scores were relatively high and clustered between 0.78 and 0.85.^8^ The highest scores were for mothers-in-law with migrant husbands (although there are very few women in this group), while the lowest were for mothers-in-law and wives with husbands in the household and daughters-in-law with migrant husbands (Table 1). The proportion of women who had attained adequacy in input in productive decisions was very high, ranging from 92 to 100%. It was lowest for wives with resident husbands and reached 100% for mothers-in-law with migrant husbands. Within each social location, it was higher for women whose husbands were migrants.

The overall proportion of adequacy on the indicator of access to and decisions on credit was the lowest of all six A-WEAI indicators – only 36% of women achieved adequacy. The highest proportions were among wives and mothers-in-law with migrant husbands, who are generally seen as better credit risks because they receive and control remittances.

The control over the use of income indicator was also high across all groups of women, averaging 95% of the women achieving adequacy. Within each group, it was higher for women whose husbands were migrants. Wives and mothers-in-law whose husbands are migrants are likely to manage their household's income and expenses in their husband's absence. Women with resident husbands had the lowest adequacy in this indicator.

The asset ownership indicator also had high levels of adequacy. The quantitative indicator is based on self-reported sole or joint ownership of a productive asset, livestock or land. It does not distinguish between legal ownership (e.g. whose name is on a land title) or the more nuanced categories of family assets, *daijo* (dowry), or *pewa*, which were discussed in the qualitative data (see Pradhan, Meinzen-Dick and Theis 2019). A higher proportion of wives and mothers-in-law with migrant husbands were adequate in this indicator compared to other categories. In addition to taking on the decision-making responsibilities, in the absence of their husbands, women are more likely to report that they own assets.

Overall, 71% of the women were members of at least one group and thus were adequate in this indicator. The probability of being in a group was highest for wives with migrant husbands and daughters-in-law with resident husbands. Mothers-in-law with resident husbands had the lowest levels of adequacy in this indicator.

Time use information was collected for the previous 24 hours. Adequacy in workload was relatively low (63% of women overall). Mothers-in-law with migrant husbands had the highest levels of adequacy in workload, (although it was not statistically significantly different from the reference category of wives with resident husbands) perhaps because they delegate the work to other household members. In contrast, wives with migrant husbands had the lowest adequacy in this indicator, since they are likely taking on productive non-domestic work as well as domestic or caregiver roles in the household. On average, women spent 9 h per day working, divided about equally between domestic and non-domestic work. There was little difference across social location groups in the amount of time spent working each day (Table 2). This is a surprising finding in light of most ethnographic literature and our own qualitative reports is that mothers-in-law are not perceived to do much work because they get their daughters-in-law to do productive non-domestic as well as domestic/care work. We return to a discussion of this finding below.

**Table 2:** Descriptive statistics of working hours by social location.

		Wife	Mother-in- law	F-statistic	p	Daughter-in-law	F-statistic	p
Total hours worked	Mean	8.99	8.91	0.09	0.76	9.04	0.07	0.79
	(SE)	(0.26)	(0.28)			(0.23)		
Productive non-domestic work hours	Mean	4.35	**4.92**	5.09	0.03	4.31	0.05	0.83
	(SE)	(0.28)	**(0.30)**			(0.30)		
Domestic/care work hours	Mean	4.67	**4.00**	15.64	0.00	4.79	0.44	0.51
	(SE)	(0.15)	**(0.17)**			(0.19)		
N		859	402			542		

Notes: Estimates are means with corresponding standard errors clustered at the VDC level. Social locations are defined as follows: Wife: no in-laws in the household; Mother-in-law: at least one daughter-in-law in the household; Daughter-in-law: at least one parent-in-law in the household. **Bold** indicates statistical significance at the p < 0.05 or lower. Domestic/care work includes time spent on shopping/getting services, weaving/sewing/textiles, cooking, domestic work, caring for children, and caring for adults.

## 4 Factors related to empowerment

The OLS regression results in Table 3 allow us to examine the associations among caste/ethnicity, social location in the household, and migration status of the husband with overall empowerment scores, controlling for other factors, including age and wealth (as proxied by the asset index). Table 4 allows us to look in more detail at how each of these factors influence each of the different dimensions of women's empowerment, by A-WEAI indicator. We first discuss the effect of caste and ethnicity overall and on each indicator, then turn to social position in the household, integrating qualitative findings that support or seemingly contradict the quantitative results.

**Table 3:** Factors influencing women's empowerment in Nepal.

	Empowerment score
*Caste reference group: Brahmin/Chhetri*	
Dalit	−0.058***
	(0.011)
Janajati	−0.037**
	(0.015)
Tarai Middle	−0.049***
	(0.012)
Muslim	−0.025
	(0.023)
Other castes	−0.041*
	(0.023)
*Social location reference group: Wife*	
Mother-in-law	−0.017
	(0.015)
Daughter-in-law	0.030**
	(0.015)
Migrant husband	0.038***
	(0.013)
Mother-in-law * Migrant husband	0.021
	(0.034)
Daughter-in-law * Migrant husband	−0.056***
	(0.020)
*Other factors*	
Age (years)	0.007***
	(0.002)
Age squared	−0.000***
	(0.000)
Years of schooling	−0.003**
	(0.001)
Child under 5 years lives in household	0.016*
	(0.010)
Child aged 5–18 years lives in household	−0.018*
	(0.010)
Number of adult women in household	0.002
	(0.006)
Number of adult men in household	0.001
	(0.005)
Asset index	0.006
	(0.005)
	
N	1803
R-squared	0.040
*Caste/ethnic group*	
Test of joint significance: F statistic	6.34
Prob > F	0.000
*Social location*	
Test of joint significance: F statistic	4.12
Prob > F	0.016

Note: OLS regressions; standard errors (in parentheses) clustered at the VDC level; * = p < 0.10; ** = p < 0.05; *** = p < 0.01.

**Table 4:** Factors influencing different dimensions of women's empowerment in Nepal, by A-WEAI indicator.

	Input in productive decisions	Asset ownership	Access to and decisions on credit	Control over use of income	Group membership	Workload
*Caste reference group: Brahmin/Chhetri*						
Dalit	1.268	0.844	0.545***	0.913	0.855	0.390***
	(0.668–2.408)	(0.404–1.764)	(0.417–0.711)	(0.462–1.804)	(0.641–1.140)	(0.298–0.509)
Janajati	0.567	0.457*	0.775	0.309***	1.013	0.807
	(0.273–1.174)	(0.197–1.061)	(0.540–1.114)	(0.151–0.635)	(0.682–1.505)	(0.554–1.175)
Tarai Middle	0.405***	0.393**	0.654***	0.653	0.566***	1.010
	(0.223–0.734)	(0.190–0.810)	(0.481–0.888)	(0.319–1.339)	(0.410–0.780)	(0.731–1.396)
Muslim	0.627	0.842	1.111	2.175	0.549**	1.010
	(0.204–1.930)	(0.182–3.885)	(0.641–1.925)	(0.280–16.904)	(0.312–0.964)	(0.559–1.825)
Other castes	0.245***	0.239***	0.636	0.241***	0.972	1.792*
	(0.103–0.584)	(0.088–0.647)	(0.361–1.118)	(0.097–0.600)	(0.529–1.787)	(0.910–3.530)
	(0.777–1.294)	(0.744–1.284)	(0.920–1.169)	(0.694–1.156)	(0.907–1.172)	(0.899–1.142)
*Social location reference group: Wife*						
Mother-in-law	1.771	1.306	0.752	1.152	0.931	0.652**
	(0.804–3.902)	(0.551–3.094)	(0.515–1.099)	(0.540–2.456)	(0.637–1.363)	(0.450–0.945)
Daughter-in-law	1.533	0.895	0.968	1.460	1.445*	1.277
	(0.746–3.151)	(0.419–1.913)	(0.675–1.387)	(0.689–3.093)	(0.972–2.150)	(0.878–1.856)
Migrant husband	1.713	4.429**	1.179	4.827***	1.428**	0.976
	(0.858–3.421)	(1.310–14.978)	(0.862–1.611)	(1.676–13.901)	(1.004–2.031)	(0.706–1.349)
Mother-in-law * Migrant husband	†	†	1.693	†	0.731	1.636
			(0.752–3.810)		(0.301–1.773)	(0.683–3.919)
Daughter-in-law * Migrant husband	0.998	0.291*	0.788	0.305*	0.441***	0.786
	(0.353–2.821)	(0.070–1.206)	(0.490–1.269)	(0.080–1.170)	(0.260–0.747)	(0.483–1.279)
*Other factors*						
Age (years)	1.074	1.039	1.166***	1.002	1.101***	1.005
	(0.985–1.171)	(0.934–1.157)	(1.097–1.239)	(0.896–1.121)	(1.042–1.163)	(0.955–1.059)
Age squared	0.999**	1.000	0.998***	1.000	0.999***	1.000
	(0.998–1.000)	(0.998–1.001)	(0.997–0.999)	(0.999–1.001)	(0.998–0.999)	(0.999–1.001)
Years of schooling	0.889***	0.962	0.985	1.002	1.032*	0.943***
	(0.834–0.947)	(0.893–1.036)	(0.953–1.018)	(0.931–1.078)	(0.996–1.071)	(0.912–0.975)
Child under 5 years in household	1.087	1.326	1.238*	0.908	0.955	1.371**
	(0.668–1.770)	(0.767–2.294)	(0.974–1.575)	(0.550–1.498)	(0.744–1.225)	(1.077–1.745)
Child aged 5–18 years in household	1.229	1.495	1.231	0.947	0.808	0.654***
	(0.748–2.018)	(0.851–2.626)	(0.939–1.613)	(0.533–1.685)	(0.612–1.067)	(0.498–0.859)
Number of adult women in household	0.842	0.781	0.988	0.916	1.103	1.057
	(0.616–1.152)	(0.557–1.095)	(0.851–1.148)	(0.666–1.260)	(0.940–1.296)	(0.910–1.227)
Number of adult men in household	1.003	0.978	1.037	0.896	1.031	1.013
	(0.777–1.294)	(0.744–1.284)	(0.920–1.169)	(0.694–1.156)	(0.907–1.172)	(0.899–1.142)
Asset index	1.496***	1.589***	0.879*	1.298*	1.026	0.941
	(1.135–1.972)	(1.160–2.178)	(0.769–1.004)	(0.973–1.733)	(0.895–1.175)	(0.826–1.073)
						
N	1772	1772	1803	1772	1803	1803
Pseudo R-squared	0.0747	0.0710	0.0429	0.0684	0.0344	0.0567
*Caste/ethnic group*						
Test of joint significance: Chi2 statistic	24.39	13.67	23.70	22.09	18.88	84.16
Prob > Chi2	0.000	0.018	0.000	0.001	0.002	0.000
*Social location*						
Test of joint significance: Chi2 statistic	2.58	0.65	2.30	0.98	4.37	9.94
Prob > Chi2	0.275	0.723	0.317	0.614	0.112	0.007

Note: Logistic regressions; 95% confidence intervals clustered at VDC level; * = p < 0.10; ** = p < 0.05; *** = p < 0.01. Social locations are defined as follows: Wife: no in-laws in the household; Mother-in-law: at least one daughter-in-law in the household; Daughter-in-law: at least one parent-in-law in the household. †Mother-in-law*Migrant husband did not vary in input in productive decisions, asset ownership and control over use of income – all women in this category were adequate in these indicators, and dropped from these regressions (N = 31). Definitions of A-WEAI indicators are provided in Appendix A.

### 4.1 Caste and ethnicity

In terms of overall empowerment score, all of the groups other than Muslim have lower empowerment scores than the Brahmin/Chhetri caste (reference category). Thus although some of the ethnographic literature cited above notes that higher caste women are subject to more restrictive gender norms, especially in terms of work, mobility, and participation in social activities, lower caste women are subject to discrimination in the home and community (Bennett 2008). Our qualitative analyses found that high caste women and women in Madhesi households (people of the plains or Tarai with culture very similar to North India) faced considerable gender discrimination within the family, including restrictions on mobility, such as not being able to visit their natal home, markets, or participate in local groups (Khalil and Mookerjee 2019). The combination of qualitative and quantitative findings suggest that, while high-caste women may be disempowered by patriarchal norms, women from low castes or other ethnic groups experience disempowerment additionally through poverty and social exclusion. The latter forms of disempowerment are better captured by A-WEAI, which is weighted toward economic empowerment and instrumental agency (“power to”), including control over assets and income, participation in decision-making, access to credit, group membership, and time (Malapit et al., 2017) but does not include freedom of movement, respect among household members, self-efficacy, or critical consciousness, which may be more important sources of disempowerment for high caste women (O'Hara and Clement 2018).^9^

The contributions of each of the six indicators to (dis)empowerment differ across the caste and ethnic groups (Table 4). Among Dalits, lower empowerment scores were driven by less access to decisions on credit and higher workload. The lower decision-making on credit is unlikely to be driven by intrahousehold restrictions, as Dalit women work for wages, which gives them more bargaining power within the household (Cameron 1995). Rather, our ethnographic data indicate the whole household is likely to lack access to credit owing to their poverty and caste status which excludes them from formal and informal credit. Dalit women are more likely to engage in wage work in agriculture compared to Brahmin/Chettri women (Acharya and Bennett 1983; Rajkarnikar 2017; Cameron 1995), increasing their overall workload.

Compared to Brahmin or Chhetri women, Janajati women were less likely to be adequate in control over use of income and asset ownership. This finding is surprising because, among all the castes, the Janajatis are considered to have the most egalitarian gender norms (Pradhan 2014; Shah 2018) and are more likely than upper caste women to work for wages, which would be expected to translate to more decision-making power.

Tarai Middle women were less likely than high caste women to be adequate in four of the six indicators: input in productive decisions, asset ownership, access to and decisions on credit, and group membership. This is consistent with our ethnographic findings and published reports that these Madhesi groups, like North Indian castes, have stricter gender norms and more inegalitarian gender relations than hill groups (Bennett et al., 2013; Morgan and Niraula, 1995; Rajkarnikar 2017; Sijapati et al., 2017).

The sample included relatively few Muslim women, which may be why there was no statistically significant difference in overall empowerment score between them and Brahmin/Chhetri women. Muslims are considered to have stricter gender norms and seclusion rules than Hindus. Muslim women were less likely to be adequate in group membership compared to Brahmin/Chettris, which is consistent with stricter seclusion of women and less mobility. Similarly, differences in empowerment scores between Brahmin/Chhetri women and women of the “other castes” category were only marginally significant, but women of other castes were less likely to be adequate in input in productive decisions, asset ownership, and control over use of income, and marginally more likely to be adequate in workload.

### 4.2 Social location and presence of husbands

The qualitative evidence lays out a strong effect of women's social location on their empowerment: young women who marry into an extended family have the least control over resources and are at the beck and call of their mothers-in-law (Pradhan, Meinzen-Dick and Theis 2019; see also Kaspar 2005; Adhikari and Hobley 2015; Rajkarnikar 2017, 2020; Sijapati et al., 2017; Singh 2016; for India see Debnath 2015; Khalil and Mookerjee 2019). The term *dukkha* is commonly used to refer to the suffering experienced in this stage of life and is a recurring theme in women's narratives in Nepal (see also March, 2002). As one 80-year old Magar widow reported of her early years as a daughter-in-law: “I did whatever my mother-in-law ordered me to do, went wherever she asked me to go. I was never allowed to go where I wanted to go. I was never allowed to do what I wanted. I could only go out freely when I wanted to urinate and defecate.”

When the extended family splits and the marital couple set up their own household, women report increased control over income and assets, greater participation in decision-making, and the ability to exercise greater agency. In a life history interview, one woman contrasted her situation while living in a joint household and after the splitting of the household. She said that she had a very hard time while she was living with her in-laws. She had to work hard, taking care of the goats, fetching water, fodder and firewood, and doing other things the whole day. But she was not properly fed, only corn and soya beans and cold rice. She could not say anything even though she was angry. “After we separated (lived in a different household), it is very convenient and easy for me now. I can eat if I want to, not eat if I do not want to, whether I work or not is in my hands.” Another woman described the difference thus: “I now cook whatever I want to … without fear. Earlier, I used to be scared … I lived under the control of my mother-in-law. I did whatever I was told to do. I was not allowed to cook what I liked.” A successful vegetable farmer reported: “After we separated [from the joint household], we consult with each other, whatever we do. Neither he nor I make decisions alone” (quoted in Pradhan, Meinzen-Dick and Theis 2019).

This pattern of greater empowerment when a couple splits off from the extended household is embedded in a local folk song, in which a young daughter-in-law is berated for breaking a water pot and returns, weeping, to her natal home. Her husband follows and coaxes her back with the promise that things will get better when they separate from the extended family:

My mother has become old, will die shortly,

Sister will get married to her home,

Remained will be my brother, I will separate him,

We both will rule our regime. (Cited in Leder et al., 2016)

These qualitative findings also speak to the notion that the husband can be a mediating presence within the household, especially when they are living with his parents. Despite greater autonomy for women in making a wide variety of decisions after the couple splits off to form a nuclear household, many women report that they still do not make final decisions about selling livestock or other produce. They explain that they want to avoid being accused of making bad decisions and to maintain harmony with their husbands—an example of inhabiting social norms of being a “good wife” (Mahmood 2005). This is consistent with the literature that reports wives having autonomy on everyday decisions, but consulting their husbands for more significant matters, including large financial matters (Sijapati et al., 2017).

Mothers-in-law are perceived as even more powerful than wives, because they have authority over daughters-in-law, to whom they can assign work (Bennett 1983). One woman's account shows how her experiences of mobility restrictions changed over her life-cycle. She recalls how as a daughter-in-law her husband's parents would not allow her to leave the homestead and she had to ask permission for everything. After separating from the joint household, her mobility increased and now as a middle-aged woman in charge of her family, she does not have to take permission from anyone, not even from her husband who works in Kathmandu. Yet she limits the mobility of her daughter-in-law, whose husband is abroad. This story illustrates how women who were passive recipients of gendered norms become its active agents as they move from a less powerful position (daughter-in-law) to a more powerful one (mother-in-law)—an example of patriarchal bargaining (Kandiyoti 1988).

Statistical analysis of factors affecting empowerment indicates that social position has a jointly significant effect on overall empowerment scores (Table 3). But when we look at individual indicators, social location only has a jointly significant effect on workload (Table 4). Yet even there, mothers-in-law are more likely than wives to be inadequate in terms of workload, which is a surprising finding that we explore in more detail below.

Wives with migrant husbands are more likely to have higher empowerment scores than wives whose husbands were resident. These findings are consistent with the literature (Kaspar 2005; Adhikari and Hobley 2015; Rajkarnikar 2017, 2020; Sijapati et al., 2017) and our ethnographic findings that both wives and mothers-in-law with migrant husbands act as de facto heads of households during their husband's absence and take on decision-making responsibilities for agricultural production and management of household affairs. Wives with migrant husbands were more likely to be adequate in asset ownership, control over use of income, and group membership. Our qualitative findings revealed that migrant husbands may ask their wives to purchase land in their wives' names, so the husband need not be there for the sale or purchase (Pradhan, Meinzen-Dick and Theis, 2019). Wives with migrant husbands also have more autonomy over use of household income, including remittances from their husbands, even though increasing availability of mobile phones and internet allows migrant husbands to be involved in household management. The positive association with adequacy in group membership could arise from several mechanisms. First, in the absence of her husband and in-laws, norms that govern mobility restrictions may be lessened, allowing women to travel to group meetings. Second, our qualitative data indicate women may join groups as a form of collective agency, participating in and contributing to the group, but also being able to seek support in times of trouble (see also Rajkarnikar 2017).

While on the whole, wives with migrant husbands may have higher empowerment scores, enjoy more freedom, autonomy and power, they also take on more responsibilities and may have higher workload and stress. As explained in the life history of one woman with a migrant husband and five children at home, after her husband migrated, she got “*sukha*” [happiness/comfort], but “When you have to do everything yourself, it becomes a burden.” (See also Adhikari and Hobley 2015; Clement et al., 2019; Clement and Sugden 2021; Kaspar 2005; Rajkarnikar 2017; Sijapati et al., 2017). In some cases, women's freedom of movement and ability to join groups after husbands migrate may be limited, either due to restrictions by in-laws or absent husbands to reduce suspicion of extramarital affairs or due to workload (see also Sijapati et al., 2017). As one woman reported, her migrant husband said “When there is a lot of work at home, if you get up and walk [away] during such work, who does [it]? The situation at home deteriorates.” Several women stated that they prefer their husbands to remain in the village rather than migrating for work because it would make their life easier or because they missed their husbands – hinting at emotional and physical ties that go beyond material conditions.

One of the mediating factors regarding husband's migration is whether he sends sufficient remittances to hire wage laborers to work in the fields (Noray 2017; World Bank/FAO 2018). Our qualitative study indicates daughters-in-law who receive remittances from their husbands may migrate to urban areas, especially if they have school-age children, to get away from in-laws and difficult life in village (see also Maharjan, 2015).

There was no difference in the overall empowerment score between wives with resident husbands and mothers-in-law with migrant or resident husbands, and only one indicator had any significant difference: mothers-in-law with resident husbands were less likely to be adequate with regard to workload than wives. We return to this issue below.

Whether a husband is a migrant or resident is highly correlated with the empowerment of daughters-in-law. Daughters-in-law with resident husbands were likely to have higher overall empowerment scores than wives with resident husbands. But daughters-in-law with migrant husbands had significantly lower empowerment scores, perhaps reflecting a lack of a husband to mediate relations with his family, as in the song cited above. The qualitative data also gave examples of in-laws physically and emotionally abusing women whose husbands were away. As Kaspar (2005:54) notes: “A husband is the connecting link between his wife and his parents,” and she depends on him to advocate for her interests. For a daughter-in-law, having a migrant husband is correlated with being less likely to have achieved adequacy in asset ownership, control over income, and group membership.

Based on statements about *dukkha* (suffering and workload) in the qualitative work, we had anticipated that daughters-in-law would be inadequate in this indicator because of high workloads. But there were no statistically significant results on time use for daughters-in-law with resident husbands compared with wives. We hypothesize that the husband's presence has a protective effect on a daughter-in-law's adequacy in workload.

When the husband is a migrant, even after controlling for other factors like caste, age, and schooling, daughters-in-law have the lowest empowerment scores of all the social location-husband's residence categories, especially in asset ownership, control over use of income and group membership. When a woman marries and moves in with her husband's family, the assets she brings to marriage may be taken to help maintain the household's expenses or pay for emergencies, or her personal assets may be kept in her parents' house until she has more control over assets (Pradhan, Meinzen-Dick and Theis, 2019). Furthermore, daughters-in-law may have limited access to the remittances their husbands send or to household-generated income, which is often managed by her in-laws (Kaspar 2005; Rajkarnikar 2017). The lack of group membership for these women does not apply to daughters-in-law with resident husbands. It may reflect greater mobility restrictions on daughters-in-law when their husbands are absent and not able to advocate for them.

Thus, the quantitative findings suggest that having a migrant husband is empowering for women in nuclear households, but disempowering if they live with their in-laws. These findings are consistent with our ethnographic findings and with the literature (Kaspar 2005; Adhikari and Hobley 2015; Rajkarnikar 2017; Singh 2016; Sijapati et al., 2017 for Nepal and Desai and Banerji 2008 in India, and Rashid 2013 in Bangladesh and other countries, e.g. Menijvar and Agadjinian 2007; Arias 2013).

#### 4.1.1 Effects of other individual and household characteristics

Finally, the relationship of empowerment with some of the other individual and household characteristics is worth noting. Increasing age was positively associated with empowerment, especially on credit and group membership. The negative effect of the age squared suggests empowerment increases at a decreasing rate along the life course. Our life history data indicates older women may become vulnerable as they become dependent on others to take care of them in their old age (see Pradhan, Meinzen-Dick and Theis 2019).^10^

The small negative coefficient on years of schooling is surprising, given prevalent literature that education is empowering for women. This finding likely reveals a cohort effect since younger women in the sample (who tend to be daughters-in-law) have had better access to education than older women.^11^ Thus schooling may be picking up some of the association of disempowerment with youth and daughter-in-law status, implying that coefficients for those variables may be conservative estimates. Years of school is low across the sample, with a mean of less than six years even for daughters-in-law.

#### 4.1.2 Workload and time agency

Based on our qualitative data and the literature, we expected to find mothers-in-law more likely to be adequate in the workload indicator, but the quantitative data on workloads do not support the notion that mothers-in-law turn over much of their work to their daughters-in-law.

One reason may be that many women are just slightly above or below the threshold for adequacy in workload (10.5 h). To address this, we explore the relationship of social location and migrant status with hours worked, rather than the binary indicator of whether they are above the threshold. Yet, the number of hours worked per day is similar across the three groups of women. The key difference is that daughters-in-law spend more of their time in domestic/care work and less in productive non-domestic work compared to wives and mothers-in-law.

We run regressions similar to those in Equation (1), using hours worked per day as the dependent variable.^12^ We then rerun a similar set of regressions, distinguishing hours spent in productive non-domestic work and those spent in domestic work. The results presented in Table 5 show no statistically significant differences across women in the different social locations.

**Table 5:** Factors influencing women's time use in Nepal.

	Total time spent on work (hours)	Time spent on non-domestic productive work (hours)	Time spent on domestic/care work (hours)
	(1)	(2)	(3)
*Caste reference group: Brahmin/Chhetri*			
Dalit	1.155*** (0.209)	1.495*** (0.211)	−0.338* (0.189)
Janajati	−0.261 (0.290)	0.047 (0.293)	−0.201 (0.261)
Tarai Middle	0.093 (0.244)	0.045 (0.246)	0.047 (0.220)
Muslim	0.628 (0.444)	0.341 (0.449)	0.267 (0.401)
Other castes	−0.723 (0.448)	−1.100** (0.453)	0.382 (0.404)
*Social location reference group: Wife*			
Mother-in-law	0.389 (0.290)	0.470 (0.293)	−0.150 (0.261)
Daughter-in-law	−0.118 (0.288)	0.267 (0.291)	−0.410 (0.259)
Migrant husband	0.030 (0.257)	0.309 (0.259)	−0.260 (0.232)
Mother-in-law * Migrant husband	−0.839 (0.667)	−0.714 (0.674)	0.023 (0.602)
Daughter-in-law * Migrant husband	−0.075 (0.383)	0.086 (0.387)	−0.096 (0.345)
Asset index	0.194* (0.102)	0.225** (0.103)	−0.041 (0.092)
*Other factors*			
Age (years)	0.029 (0.040)	0.094** (0.041)	−0.063* (0.036)
Age squared	−0.000 (0.000)	−0.001*** (0.000)	0.001 (0.000)
Years of schooling	0.072*** (0.026)	−0.115*** (0.026)	0.184*** (0.024)
Child under 5 years lives in household	−0.462** (0.188)	−0.234 (0.190)	−0.212 (0.170)
Child aged 5–18 years lives in household	0.518** (0.205)	0.445** (0.208)	0.087 (0.185)
Number of adult women in household	−0.083 (0.117)	−0.104 (0.119)	0.018 (0.106)
Number of adult men in household	0.019 (0.094)	0.090 (0.095)	−0.049 (0.085)
N	1803	1803	1803
R-squared	0.057	0.088	0.066
*Caste*			
Test of joint significance: F statistic	11.65	18.26	1.66
Prob > F	0.000	0.000	0.142
*Social location*			
Test of joint significance: F statistic	1.35	1.36	1.25
Prob > F	0.259	0.257	0.288

Note: OLS regressions; standard errors clustered at the VDC level; * = p < 0.10; ** = p < 0.05; *** = p < 0.01.

The lack of significant quantitative differences in workload differs from the qualitative narratives, in which women recall the long hours they had to work as daughters-in-law as including productive work – tending to animals, collecting fuel, water, fodder and working in the fields. It is possible that some of this productive non-domestic work was intermingled with domestic/care tasks and reported as such in the time use surveys. In the qualitative reports on women's time, they often indicate that they were cooking and tending animals in the same block of time, for example: “Fed buffalo, cooked and fed snacks for guests and self.“.

While the qualitative findings are not necessarily representative because of the limited sample size, they provide important insights into how time use and empowerment are related. The issue seems to be not only that daughters-in-law feel they are doing many more hours of work, but more importantly that they have little or no control over their work. In other words, they have less autonomy over work (and many other things, including mobility) and thus less agency. The mothers-in-law assign the household tasks and ensure that they are done. The daughter-in-law does not choose what tasks she does or when she does them. The qualitative work suggests that daughter-in-law's lack of agency over their work is at least partially responsible for their complaints. They are assigned the least pleasant tasks and may be scolded or sometimes even beaten if they are not done according to their mother-in-law's demands. Our qualitative data on what women did often indicates daughters-in-law multitasking, supporting the idea of intensity of work. It is also the case that in the quantitative data, each woman is providing information on her own time use and it may be that mothers-in-law are working more hours than is perceived by the daughters-in-law. These findings suggest that we need to consider more than just hours worked as an indicator of empowerment, but also to consider agency over work.

We identify four caveats to the interpretation of our quantitative findings. There are few mothers-in-law with migrant husbands in our sample. This may be because the average age of migrants working abroad is estimated to be 30 (Sharma et al., 2014) or 31 (World Bank/FAO 2018). In addition, our quantitative analysis is limited to the A-WEAI indicators of empowerment. Indicators such as freedom of movement or respect among household members would be useful to further unpack the complex relationship between social location and husband's residence status. Third, although we examine the effects of caste/ethnicity, social location and husband's residence status in separate analyses, we expect that caste/ethnicity also interacts with social location and husband's residence status but are unable to analyze this hypothesis further due to limited sample size. Finally, the analysis does not account for the relationship between caste/ethnicity and migration status, especially if we expect that economic opportunities may positively influence individuals of lower castes and ethnic groups to migrate for work compared to individuals of higher castes.

## 5 Conclusions

Combining qualitative and quantitative evidence on women's empowerment provides important insights—and some surprises—regarding the relationships with caste and ethnicity and with social location within the household and husband's status as a migrant. The qualitative findings point out key areas that are missed in the quantitative analysis using the A-WEAI. This has implications for the ongoing work of how to quantitatively measure women's empowerment but also points to the importance of mixed methods research.

First, while caste and ethnicity are important factors affecting women's empowerment, the relationships are complex. The A-WEAI indicators suggest that Dalit and Janajati women are less empowered than Brahmin/Chhetri women, which does not match the ethnographic literature focusing on patriarchal norms of high castes, but is consistent with literature on on the effects of poverty and marginalization of Dalit and Janajati men and women in Nepal (e.g. Bennett 2008; Leder and Sachs 2019; Nightingale 2011; Sugden et al., 2014; Tamang 2011) The patterns differ across the various indicators of empowerment and the qualitative findings suggest that the A-WEAI may be missing components of empowerment that are relevant in rural Nepal. For example, Brahmin/Chhetri women are often considered disempowered because of the social restrictions on their mobility and participation in civil society, but this is not well captured in the A-WEAI. Nonetheless, our results indicate that Dalit and Janajati women are disempowered due to poverty and social exclusion, rather than primarily through patriarchal gender norms. Further analysis of men's as well as women's levels of empowerment, and more extensive measures of empowerment^13^ can help to distinguish between these sources of disempowerment, and help guide interventions to address the appropriate root causes. Disempowerment through poverty may manifest differently than disempowerment through patriarchy.

Second, the findings suggest that the migration status of the husband plays a role in the levels of women's empowerment. Wives in nuclear households are more empowered if their husband is a migrant; they may have higher workloads, but they also have more control over agricultural production and income. For daughters-in-law, their empowerment is higher when their husband is resident, which may be because he is able to mediate the influence of his family. In particular, when he is resident, she is more likely to be adequate in both group membership and workload. The qualitative evidence suggests that the dynamic story may be more complex, depending on the relations within the family and the level of remittances. Daughters-in-law may be willing or eager for their husbands to migrate, even though they will suffer living under their mother-in-law's control, because the income that the migrant husband earns may allow the couple to then separate and become an independent household. Not all migration is successful; some men are not able to save money or send remittances, which then leaves the women potentially worse off.

Finally, the seemingly contradictory qualitative and quantitative results on the relationship of social location and time use suggest the importance of considering agency over time, rather than just the hours worked. The narrative among daughters-in-law is not only that that they work hard, but also that they have no control over their work and receive few of the benefits, complaining that they are poorly treated and not well fed. This narrative is not simply about the hours worked, but also, perhaps more significantly, about agency over their labor and control over the outputs. The complaint that they work particularly hard is not reflected in the quantitative data on hours worked; the hours worked reported by daughters-in-law are only marginally higher than those reported by mothers-in-law. But the time use data doesn't capture how hard they worked on a particular task nor how they felt about it. A daughter-in-law working for an hour with her mother-in-law standing over her telling her to work harder and faster to get on to the next task will be perceived very differently from the hour that the mother-in-law worked as she will have had more choice over which tasks to do and may be able to work more leisurely. More nuanced ethnographic studies, such as participant observation, can shed light on these issues.

The story of women's empowerment in Nepal—as elsewhere—is a dynamic one and will continue to change. It will be important to continue to analyze these changes over time and mixed methods approaches will be critical to fully understanding what is happening.

## Author statement

Ruth Meinzen-Dick: Conceptualization, Methodology, Writing -- original draft, Writing-review & editing, Supervision, Project administration, and Funding acquisition. Cheryl Doss: Conceptualization, Methodology, Writing -- original draft, Writing – review & editing. Audrey Pereira: Formal analysis, Writing -- original draft, Writing – review & editing. Rajendra Pradhan: Conceptualization, Methodology, Investigation, Writing – original draft, Writing – review & editing.

## Figures and Tables

**Table:** 

Domain	Indicator (Weight)	A-WEAI definition
Production	Input in productive decisions (1/5)	Adequate if individual participates in and makes decisions, has input in decisions, or feels she could make decisions (if desired) about at one agricultural activity
Resources	Ownership of assets (2/15)	Adequate if household owns at least one productive asset, livestock or land, and the respondent (individually) has at least some ownership of an asset*
	Access to and decisions about credit (1/15)	Adequate if individual makes decisions about at least one source of credit accessed by her household
Income	Control over use of income (1/5)	Adequate if individual participates in and has input in decisions about income generated from an activity or she/he makes decisions, has input in decisions, or feels she/he could make decisions (if desired) about employment or major household expenditures
Leadership	Group member (1/5)	Adequate if individual is an active member of at least one group
Time	Workload (1/5)	Adequate if individual worked fewer than 10.5 h during the previous day.

**Table:** 

	Wife				Mother-in-law				Daughter-in-law			
	Brahmin/Chhetri	Dalit	Janajati	Tarai Middle	Brahmin/Chhetri	Dalit	Janajati	Tarai Middle	Brahmin/Chhetri	Dalit	Janajati	Tarai Middle
Age (years)	40.43	40.02	41.09	39.54	53.28	51.86	51.45	50.67	29.65	30.30	28.30	29.62
	(0.72)	(0.64)	(1.28)	(0.87)	(0.80)	(0.76)	(1.18)	(1.13)	(0.66)	(0.60)	(1.21)	(0.85)
Years of schooling	4.03	2.15	1.62	1.68	0.99	0.52	0.31	0.11	7.76	5.30	3.00	3.84
	(0.29)	(0.19)	(0.30)	(0.23)	(0.23)	(0.12)	(0.19)	(0.06)	(0.36)	(0.28)	(0.53)	(0.45)
Number of adult women in HH	1.29	1.20	1.23	1.19	2.65	2.75	2.59	2.51	2.47	2.44	2.25	2.16
	(0.04)	(0.03)	(0.05)	(0.04)	(0.09)	(0.09)	(0.15)	(0.09)	(0.07)	(0.06)	(0.10)	(0.09)
Number of adult men in HH	1.56	1.42	1.36	1.33	2.99	2.91	2.90	2.82	2.32	2.34	2.25	2.30
	(0.06)	(0.04)	(0.06)	(0.05)	(0.10)	(0.09)	(0.16)	(0.10)	(0.09)	(0.09)	(0.18)	(0.11)
Child under 5 years lives in HH	0.13	0.22	0.23	0.21	0.49	0.43	0.55	0.60	0.49	0.47	0.57	0.45
	(0.02)	(0.02)	(0.04)	(0.03)	(0.05)	(0.04)	(0.07)	(0.05)	(0.04)	(0.04)	(0.07)	(0.05)
Child aged 5–18 years lives in HH	0.72	0.82	0.75	0.77	0.55	0.88	0.86	0.78	0.74	0.85	0.79	0.87
	(0.03)	(0.02)	(0.04)	(0.03)	(0.05)	(0.03)	(0.05)	(0.05)	(0.04)	(0.03)	(0.06)	(0.03)
Husband is migrant (any)	0.27	0.34	0.25	0.30	0.05	0.09	0.12	0.08	0.44	0.48	0.45	0.38
	(0.03)	(0.03)	(0.04)	(0.03)	(0.02)	(0.02)	(0.05)	(0.03)	(0.04)	(0.04)	(0.07)	(0.05)
Asset index	0.01	−0.28	−0.59	−0.57	0.60	0.29	−0.03	−0.07	0.36	0.37	−0.33	0.03
	(0.05)	(0.04)	(0.08)	(0.06)	(0.08)	(0.07)	(0.14)	(0.10)	(0.07)	(0.07)	(0.15)	(0.09)
***Empowerment outcomes***												
Empowerment score (0–1)	0.83	0.79	0.80	0.77	0.83	0.75	0.81	0.79	0.84	0.79	0.77	0.80
	(0.01)	(0.01)	(0.02)	(0.01)	(0.02)	(0.01)	(0.02)	(0.02)	(0.01)	(0.01)	(0.03)	(0.02)
Access to and decisions on credit	0.47	0.36	0.39	0.37	0.31	0.23	0.31	0.37	0.43	0.29	0.45	0.32
	(0.03)	(0.03)	(0.05)	(0.04)	(0.05)	(0.03)	(0.07)	(0.05)	(0.04)	(0.03)	(0.07)	(0.05)
Group membership	0.77	0.73	0.71	0.56	0.70	0.63	0.75	0.63	0.80	0.76	0.75	0.70
	(0.03)	(0.03)	(0.05)	(0.04)	(0.05)	(0.04)	(0.06)	(0.05)	(0.03)	(0.03)	(0.06)	(0.05)
Control over use of income	0.97	0.96	0.89	0.94	0.97	0.96	0.94	0.93	0.97	0.97	0.83	0.96
	(0.01)	(0.01)	(0.03)	(0.02)	(0.02)	(0.02)	(0.03)	(0.03)	(0.02)	(0.01)	(0.05)	(0.02)
Asset ownership	0.97	0.98	0.94	0.94	0.99	0.98	0.98	0.93	0.97	0.95	0.89	0.91
	(0.01)	(0.01)	(0.02)	(0.02)	(0.01)	(0.01)	(0.02)	(0.03)	(0.02)	(0.02)	(0.04)	(0.03)
Input in productive decisions	0.94	0.96	0.94	0.89	0.98	0.99	0.94	0.93	0.95	0.97	0.89	0.91
	(0.02)	(0.01)	(0.02)	(0.02)	(0.01)	(0.01)	(0.03)	(0.03)	(0.02)	(0.01)	(0.04)	(0.03)
Workload	0.67	0.50	0.70	0.72	0.74	0.44	0.69	0.73	0.70	0.50	0.66	0.73
	(0.03)	(0.03)	(0.05)	(0.03)	(0.04)	(0.04)	(0.07)	(0.05)	(0.04)	(0.04)	(0.07)	(0.04)
*Time use*												
Time spent on work (total)	8.85	9.80	8.12	8.54	8.21	9.80	8.13	8.46	8.88	9.73	8.20	8.74
	(0.22)	(0.19)	(0.35)	(0.24)	(0.35)	(0.30)	(0.48)	(0.39)	(0.26)	(0.25)	(0.50)	(0.29)
Productive non-domestic work (hours/day)	3.94	5.33	4.06	3.76	3.99	6.07	4.25	4.53	3.43	5.42	3.69	4.14
	(0.21)	(0.21)	(0.33)	(0.25)	(0.29)	(0.32)	(0.47)	(0.34)	(0.24)	(0.27)	(0.50)	(0.30)
Domestic/care work (hours/day)	4.95 (0.20)	4.48	4.27	4.79	4.22	3.76	3.88	3.93	5.44	4.36	4.64	4.68
		(0.18)	(0.25)	(0.22)	(0.29)	(0.22)	(0.38)	(0.33)	(0.29)	(0.24)	(0.42)	(0.27)
N	214	298	102	180	97	150	51	83	147	193	53	104

## Appendix A Definitions and weighting of A-WEAI indicators

Note: Full details on the A-WEAI are available in Malapit et al. (2017); *Ownership of assets indicator deviates from A-WEAI.

## Appendix B Descriptive statistics of women in the sample, by social location and caste (means and standard errors)

Note: Estimates are means with corresponding standard errors clustered at the VDC level in parentheses. Social locations are defined as follows: Wife: no in-laws in the household; Mother-in-law: at least one daughter-in-law in the household; Daughter-in-law: at least one parent-in-law in the household. The empowerment score is the weighted average of the six binary A-WEAI indicators provided in Appendix A. Domestic/care work includes time spent on shopping/getting services, weaving/sewing/textiles, cooking, domestic work, caring for children, and caring for adults.

## Footnotes

1 The BASIS-funded evaluation conducted by University of Georgia, Montana State University, Nepā School of Social Sciences and Humanities, and IFPRI was designed as a randomized control trial consisting of three treatment arms and one control group. The intervention package composed of varying combinations the following: group formation, cornerstones trainings, technical trainings, and livestock (goats). The study received ethical approval from Institutional Review Board of the International Food Policy Research Institute, Washington DC (IRB number 00007490). Because this analysis is a cross-sectional exploration of the association between caste, women's social location in the household, and husband's migration on women's empowerment, we do not analyze by treatment arm. The impact evaluation results of this RCT can be found in Janzen et al. (2018).

2 Because comparable data were not collected from husbands, we cannot compute empowerment scores for men or analyze data on gender parity within the household, which is another component of the A-WEAI.

3 These are grouped as follows: Brahmin/Chhetri (Brahman-Hill, Chhetree, Thakuri), Dalit (Damai/Dholi, Kami, Sarki, Chamar/Harijan/Ram, Dusadh/Pasawan/Pasi, Khatwe, Lohar, Musahar, Tatma/Tatwa), Janajati (Chepang, Danuwar, Darai, Ghart/Bhujel, Gurung, Kamal, Magar, Majhi, Newar, Rai, Temang, Tharu), Tarai Middle (Bin, Gaderi/Bhedhar, Kalwar, Kathbaniyan, Koiri/Kushwaha, Kumhar, Kumi, Sudhi, Teli, Yadav).

4 Adequacy definitions are available in Appendix A.

5 The asset index was created using a standardized inverse-covariance weighted summary index (Anderson 2008).

6 Details on research design are available in Janzen et al. (2018)..

7 Our sample is not representative of the caste/ethnic groups overall in Nepal which are: Brahmin/Chhetri 31.2%, Dalits 12.6%, Janajati 35%, Terai middle caste 14.5%, Muslims 4.4% and others 0.3%. Therefore, our sample overrepresents the Brahmin/Chhetri, Terai middle caste, Dalit, and other groups, while underrepresenting the Janajati. This non-representation is primarily a function of the areas in which we were working.

8 By contrast, O'Hara and Clement (2018) report that the Market Access and Water Technology for Women baseline had a WEAI score of 0.711 and the Suaahara project baseline a score of 0.604. The higher A-WEAI scores for the Heifer project reflects characteristics of the Heifer project beneficiaries (and similar women from the control), especially where group membership and control over at least some assets is key to participation.

9 The project-level Women's Empowerment in Agriculture Index (pro-WEAI) includes additional indicators for autonomy in income, self-efficacy, attitudes about intimate partner violence, respect among household members, visiting important locations, and membership in influential groups (Malapit et al., 2019).

10 Analyzing nationally representative data, Rakjanikar (2017) similarly finds decision-making and workload increases with age then declines, generally after 40.

11 The correlation between age and schooling is −0.5175. The correlationd between schooling and being a daughter-in-law is 0.4085, but −0.3180 for being a mother-in-law and −0.110 for being a wife. Multicollinearity estimates were low.

12 Derived from a detailed time allocation module of activities reported during the previous 24 hours.

13 The latest of the WEAI family of indiators, the pro-WEAI, does include some of these additional indicators of empowerment(see Malapit et al., 2019).

## References

[bib1] Acharya M., Bennett L. (1983). The Status of Women in Nepal.

[bib2] Adhikari J., Hobley M. (2015). Everyone is leaving. Who will sow our fields? The livelihood effects on women of male migration from Khotang and Udaypur Districts, Nepal, to the Gulf countries and Malaysia. HIMALAYA.

[bib3] Alkire S., Meinzen-Dick R.S., Peterman A., Quisumbing A.R., Seymour G., Vaz A. (2013). The women's empowerment in agriculture index. World Dev..

[bib4] Allendorf K. (2007). Do women's land rights promote empowerment and child health in Nepal?. World Dev..

[bib5] Anderson M. (2008). Multiple inference and gender differences in the effects of early intervention: a reevaluation of the Abecedarian, Perry preschool, and early training projects. J. Am. Stat. Assoc..

[bib6] Bennett L., Dani A.A., de Haan A. (2008). Inclusive States: Social Policy and Structural Inequalities.

[bib55] Bennett L., Thapa D., Sijapati B. (2013). Gender and Social Exclusion in Nepal: Update.

[bib8] Cameron M.M. (1995). Transformations of gender and caste divisions of labor in rural Nepal: land, hierarchy, and the case of untouchable women. J. Anthropol. Res..

[bib9] Carr E.R., Thompson M.C. (2014). Gender and climate change adaptation in agrarian settings: current thinking, new directions, and research frontiers. Geography Compass.

[bib10] Clement F., Sugden F. (2021). Unheard vulnerability discourses from Tarai-Madhesh, Nepal. Geoforum.

[bib11] Clement F., Buisson M.C., Leder S., Balasubramanya S., Saikia P., Bastakoti R., van Koppen B. (2019). From women's empowerment to food security: revisiting global discourses through a cross-country analysis. Global Food Security.

[bib12] Colfer C.J.P., Sijapati Basnett B., Ihalainen M. (2018).

[bib13] Debnath S. (2015). The impact of household structure on female autonomy in developing countries. The. J. Dev. Stud..

[bib14] Desai S., Banerji M. (2008). Negotiated identities: male migration and left-behind wives in India. J. Popul. Res..

[bib15] Freire P. (1970).

[bib16] Government of Nepal, UNDP (United National Development Program) (2014).

[bib17] Janzen S., Magnan N., Sharma S., Thompson W.M. (2016).

[bib18] Janzen S., Magnan N., Sharma S., Thompson W.M. (2018). Short-term impacts of a pay-it-forward livestock transfer and training program in Nepal. *AAEA Papers and* Proceedings.

[bib19] Jayachandran S., Biradavolu M., Cooper J. (2021). https://faculty.wcas.northwestern.edu/%7Esjv340/measuring_womens_agency.pdf.

[bib20] Kabeer N. (1999). Resources, agency, achievements: reflections on the measurement of women's empowerment. Dev. Change.

[bib21] Kandiyoti D. (1988). Bargaining with patriarchy. Gend. Soc..

[bib22] Kaspar H. (2005).

[bib23] Khalil U., Mookerjee S. (2019). Patrilocal residence and women's social status: evidence from South Asia. Econ. Dev. Cult. Change.

[bib24] Leder S., Sachs C.E., Sachs C. (2019). Gender, Agriculture and Agrarian Transformations.

[bib25] Leder S., Das D., Reckers A., Karki E. (2016).

[bib27] Maharjan R.M. (2015). Emigrants’ migrant wives: linking international migration and internal migration. Stud. Nepali History Soc. 20.

[bib26] Maharjan A., Bauer S., Knerr B. (2012). Do rural women who stay behind benefit from male out-migration? A case study in the hills of Nepal. Gend. Technol. Dev..

[bib28] Mahmood S. (2005).

[bib29] Malapit H.J., Pinkstaff C., Sproule K., Kovarik C., Quisumbing A.R., Meinzen-Dick R.S. (2017). http://ebrary.ifpri.org/cdm/ref/collection/p15738coll2/id/131231.

[bib30] Malapit H., Quisumbing A., Meinzen-Dick R., Seymour G., Martinez E.M., Heckert J., Rubin D., Vaz A., Yount K.M., the Gender Agriculture Assets Project Phase 2 (GAAP2) Study Team (2019). Development of the project-level women’s empowerment in agriculture index (pro-WEAI). World Dev..

[bib31] March K. (2002).

[bib32] Meinzen-Dick R.S., Rubin D., Marlène E., Mulema A.A., Myers E. (2019). http://ebrary.ifpri.org/cdm/singleitem/collection/p15738coll2/id/133060.

[bib34] Morgan S.P., Niraula B.B. (1995). Gender inequality and fertility in two Nepali villages. Popul. Dev. Rev..

[bib35] Nightingale A.J. (2011). Bounding difference: intersectionality and the material production of gender, caste, class and environment in Nepal. Geoforum.

[bib36] Noray S.G. (2017).

[bib37] O'Hara C., Clement F. (2018). Power as agency: a critical reflection on the measurement of women's empowerment in the development sector. World Dev..

[bib38] Pradhan R. (2014). Paper Presented at the Nepal and the Himalaya Conference, Kathmandu.

[bib39] Pradhan R., Meinzen-Dick R., Theis S. (2019). Property rights, intersectionality, and women's empowerment in Nepal. J. Rural Stud..

[bib40] Rajkarnikar P.J. (2017).

[bib41] Rajkarnikar P.J. (2020). Male migration and women's decision-making in Nepal. Rev. Econ. Househ..

[bib42] Rashid S.R. (2013). Bangladeshi women's experiences of their men's migration: rethinking power, agency and subordination. Asian Surv..

[bib43] Rowlands J. (1995). Empowerment examined. Dev. Pract..

[bib44] Satyal Pravat P., Arora Vibha, Jayaram N. (2013). Routing Democracy in the Himalayas: Experiments and Experiences.

[bib45] Shah S. (2018).

[bib46] Sharma S., Pandey S., Pathak D., Sijapati-Basnett B. (2014).

[bib47] Sijapati B., Lama A.S., Baniya J., Rinck J., Jha K., Gurung A. (2017).

[bib48] Singh A. (2016). Rethinking empowerment: gender identities, agency, and women's empowerment discourse and practice in Nepal. Stud. Nepali Hist. Soc..

[bib49] Sugden F., Maskey N., Clement F., Ramesh V., Philip A., Rai A. (2014). Agrarian stress and climate change in the Eastern Gangetic Plains: gendered vulnerability in a stratified social formation. Global Environ. Change.

[bib51] Tamang S., Viswesaran K. (2011). Perspectives on Modern South Asia: A Reader in Culture, History, and Representation.

[bib52] World Bank/DFID (2006).

[bib53] World Bank/FAO (2018).

[bib54] Yount K.M., VanderEnde K.E., Dodell S., Cheong Y.F. (2016). Measurement of women's agency in Egypt: a national validation study. Soc. Indicat. Res..

